# Challenges for China's medical education in the coming post-COVID-19 era

**DOI:** 10.1093/nsr/nwaa168

**Published:** 2020-07-28

**Authors:** Li Shao, Weijie Zhao

**Affiliations:** Department of Education of Shanghai Jiao Tong University School of Medicine; NSR news editor, based, Beijing

## Abstract

The year of 2020 has been overshadowed by the COVID-19 pandemic. Medical workers throughout China have played critical roles in battling severe acute respiratory syndrome-coronavirus 2 (SARS-CoV-2) and saving lives. The whole of society has now fully realized the significance of medical workers and many began to think about medical education in China: How can we further improve medical education for the next generation of clinicians, medical scientists, nurses, public-health workers and administrators related to medical care, so that they are well prepared to meet societal needs for medical care in the ever-changing world? In this panel discussion, medical-education experts from several prominent medical schools in China gathered to discuss the reform and future development of China's medical education.

Xiang Chen

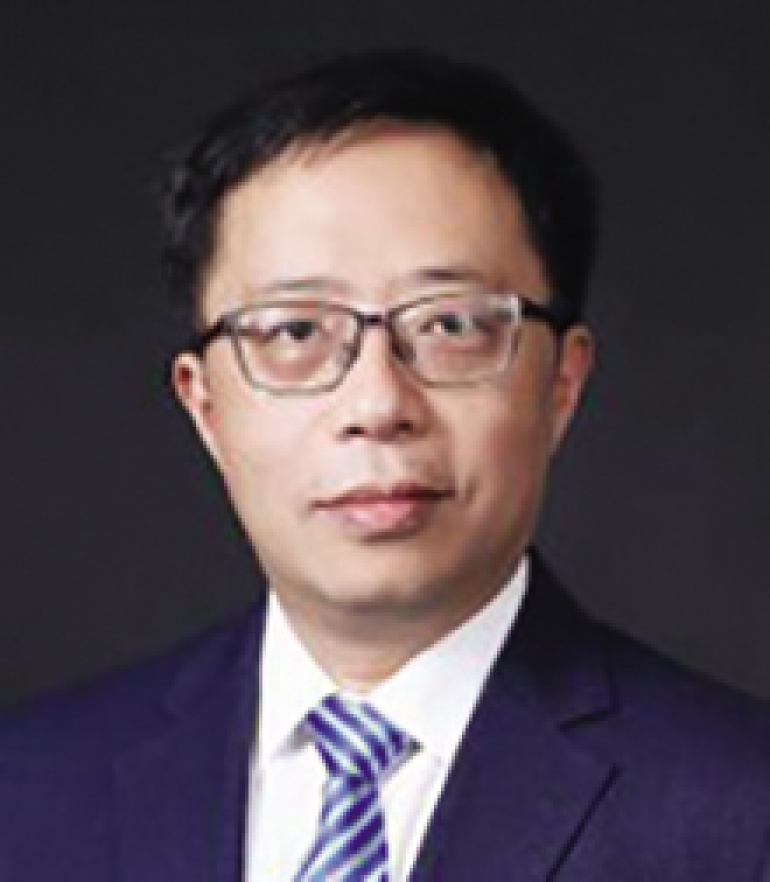

President of Xiangya School of Medicine, Vice President of Central South University, Changsha, China

Baorong Chi

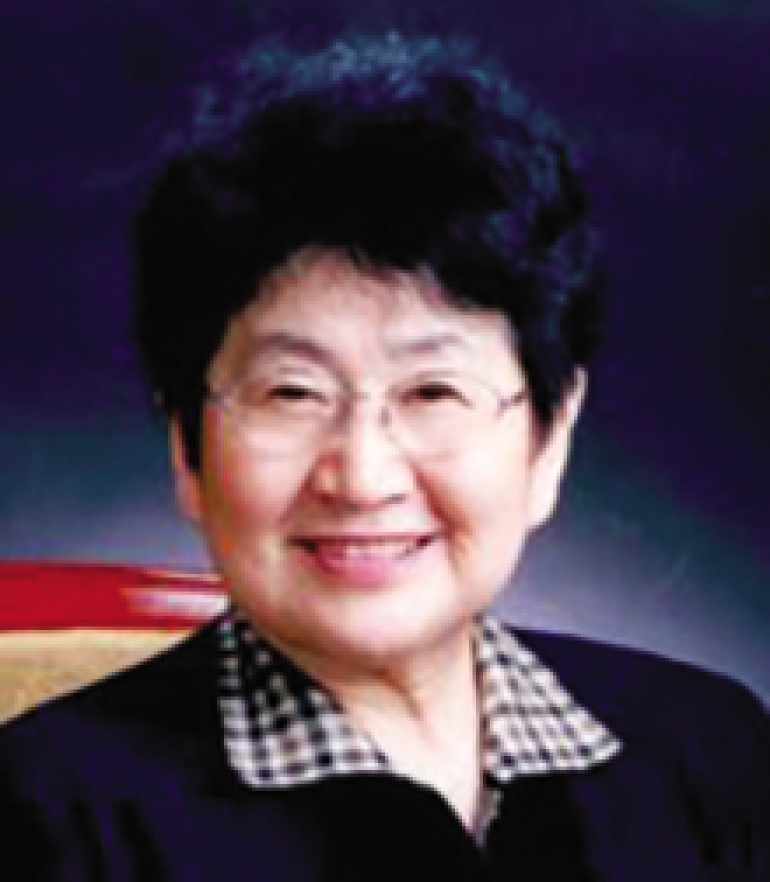

Professor of Norman Bethune Health Science Center of Jilin University, Changchun, China

Yiqun Hu

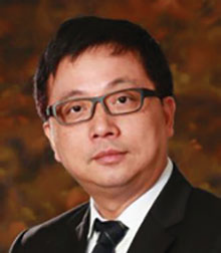

Vice Chancellor of Shanghai Jiao Tong University School of Medicine, Shanghai, China

Yang Ke

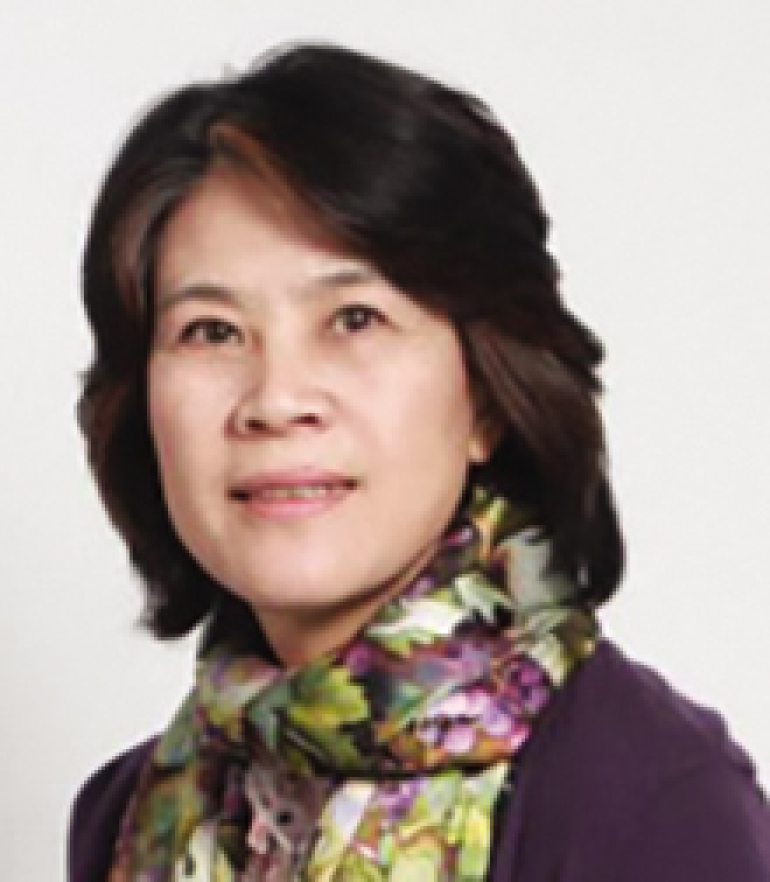

Professor of Peking University Health Science Center, former Vice President of Peking University, Beijing, China

Ming Kuang

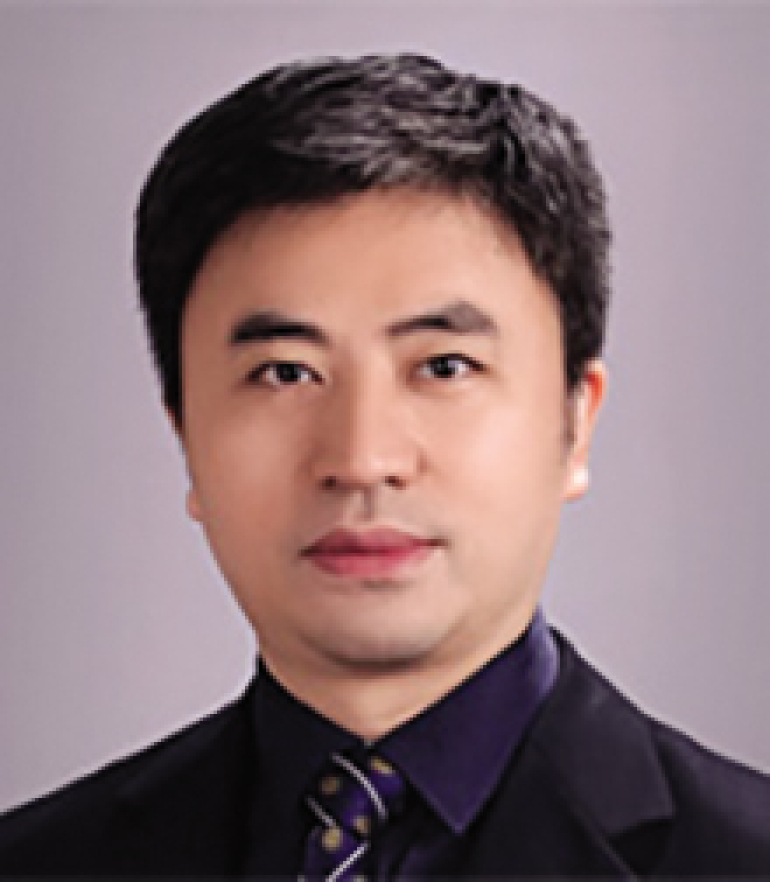

Vice President of Zhongshan School of Medicine, Sun Yat-sen University, Guangzhou, China

Mengfeng Li

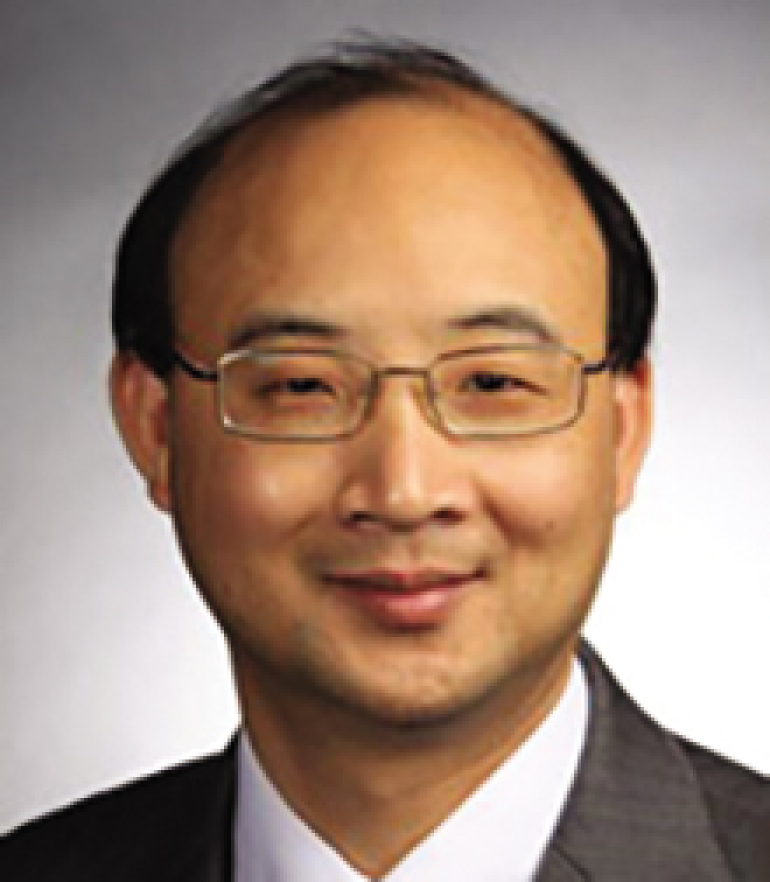

President of Southern Medical University, Guangzhou, China

Hongbing Shen

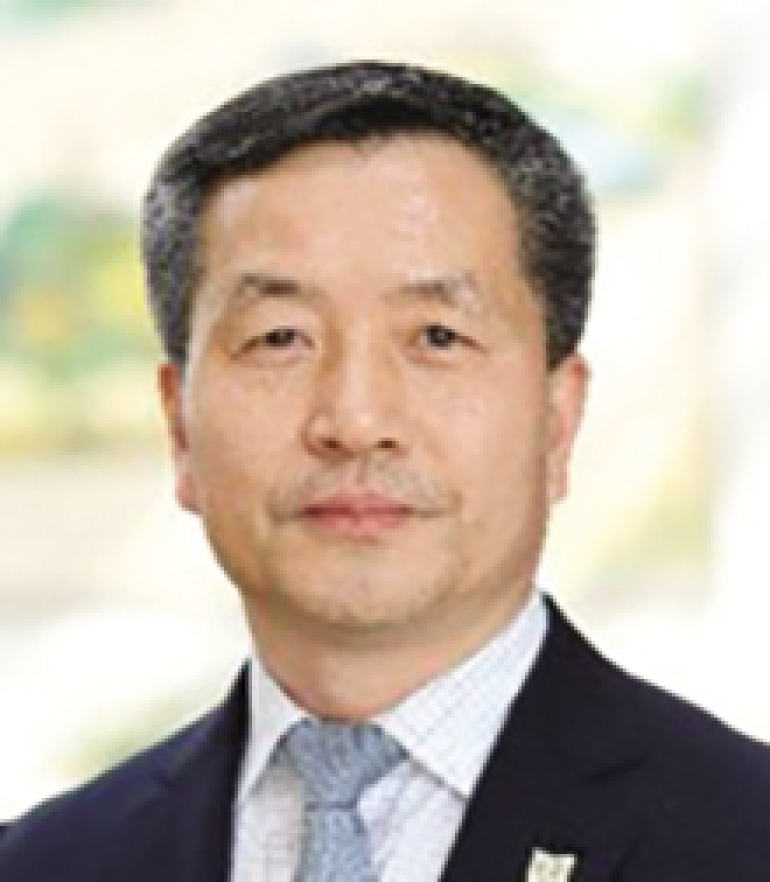

President of Nanjing Medical University, Nanjing, China

Xuehong Wan

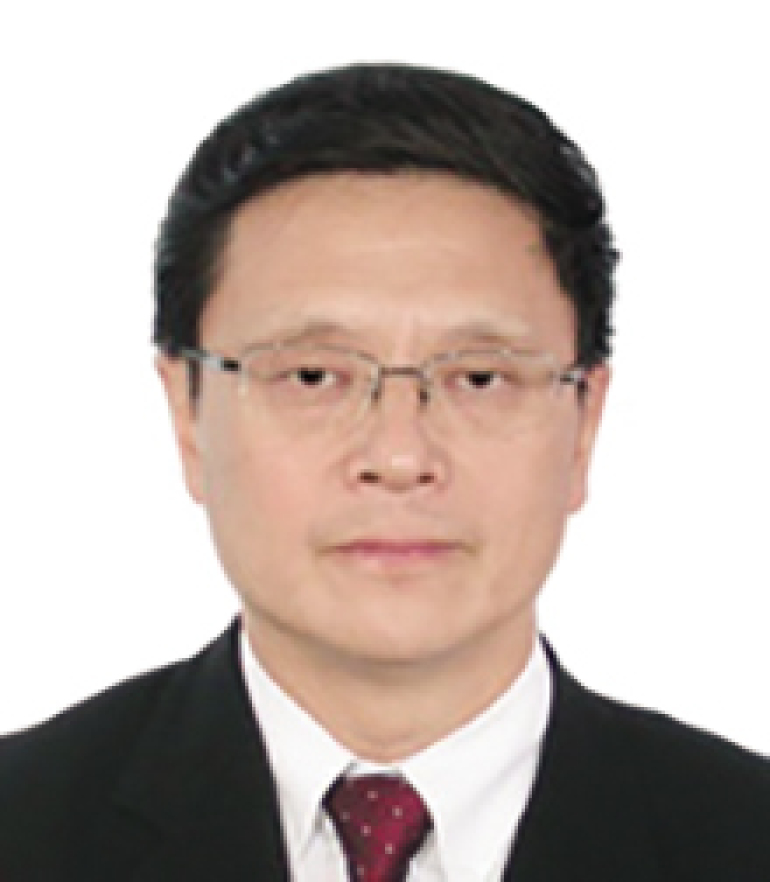

Professor of West China Medical Center, Vice President of Graduate School of Sichuan University, Chengdu, China

Hong Yan

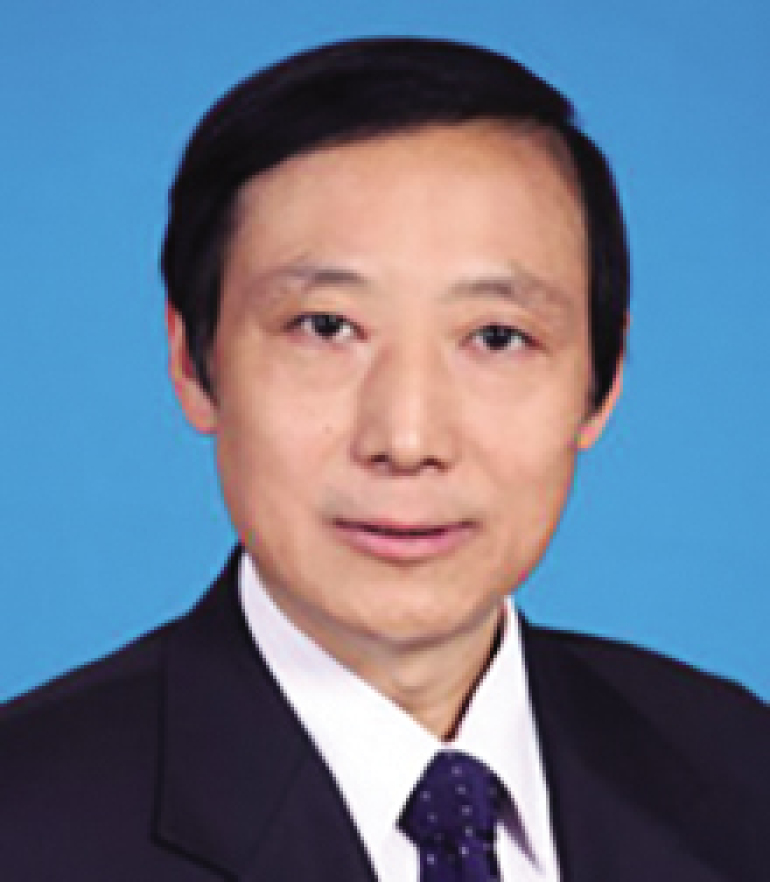

President of Xi’an Jiaotong University Health Science Center, Vice President of Xi’an Jiaotong University, Xi’an, China

Guoqiang Chen (Chair)

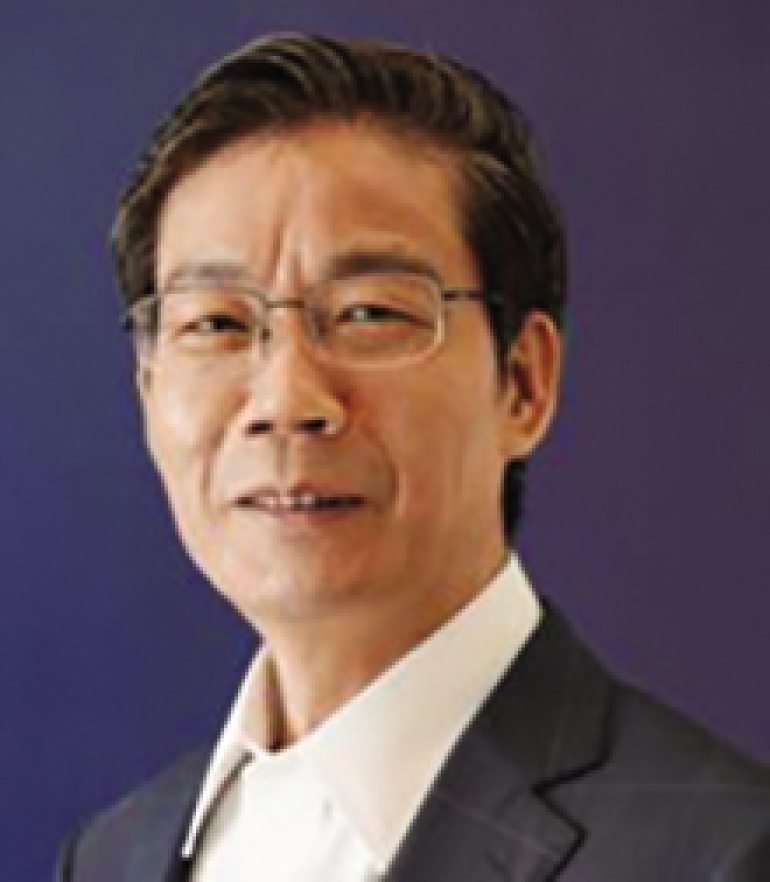

Chancellor of Shanghai Jiao Tong University School of Medicine, Vice President of Shanghai Jiao Tong University, Shanghai, China

**GQ Chen:** The COVID-19 pandemic has been the biggest public-health emergency since the founding of the People's Republic of China. The entire country was mobilized to fight the disease. Around 42 600 Chinese medical workers joined this battle. They bravely headed to the front line and saved many lives in Wuhan, Hubei province and all over China. Many heroic stories highlighted the spirit of these workers—the unyielding resolve to save lives with all their strength and love.

I believe that most people have never before better realized that medical workers are so precious and should be fully respected, and medical science should be highly valued as a science that can save the nation. Also, further reinforcement of medical education, which forms the basis for the medical and healthcare system, should be put into society's immediate agenda in the post-COVID-19 era.

Medical education in China has been greatly improved over the past decades. But, in order to step up further, we should first objectively recognize the existing problems, especially those exposed during the pandemic. That is what we are going to discuss today: the problems and the possible solutions.

## THE IMPORTANCE OF PREVENTIVE MEDICINE

**GQ Chen:** The object of medicine is humans. Humans are complicated both as an animal species and as members of civilization. So, medical science is a combination of natural science, social science and the humanities. It is the most logical one among all social sciences and the most humanistic one in all natural sciences.

Medical science includes lots of aspects, including clinical medicine, basic biomedical research, preventive medicine, nursing, medical technology, medical humanities, medical sociology and health economics. Medical science is the entirety of all these aspects and its integrity and particularity should be respected.

**BR Chi:** That is right. We should respect and protect both the integrity and the particularity of medical science. The COVID-19 pandemic exposed the problem that our public-health system had paid much more attention to treatment than prevention. We do not have enough public-health workers. On the one hand, it is hard to cultivate qualified public-health workers; on the other hand, they are not fully respected by society.

Perhaps we should readjust the proportion of students in different disciplines of medical schools and teach more about serious infectious diseases in order to cultivate medical talents with high professional competency. And, of course, the government should better support public-health workers.

**HB Shen:** The entire society, not only the medical schools, tends to pay more attention to treatment than prevention, resulting in the separation of public-health/preventive medicine and clinical medicine in an almost parallel manner. We have made efforts: clinical-medicine students do have to take several public-health courses such as epidemiology and health statistics, but these courses are usually not taken seriously by both student and teacher. Furthermore, the courses on preventive medicine are too theoretical, with insufficient attention on practical hands-on education on the scene or in disease-control centers. Students are also far from competent in understanding issues relating to health policy, epidemiological information and health management.

The entire society … tends to pay more attention to treatment than prevention, resulting in the separation of public-health/preventive medicine and clinical medicine in an almost parallel manner.

—Hongbing Shen

Considering this situation, the next step should focus on merging the teaching of clinical and preventive medicines. Teachers from both disciplines should engage more effort into the education of each other's students, in order to truly broaden the knowledge base of both groups of students and improve their practical skills. There is a long way ahead in implementing this reform.

## CULTIVATE ALL TYPES OF MEDICAL TALENTS WITH STRONG PROFESSIONAL COMPETENCY

**GQ Chen:** Clinical or preventive, the major task is to cultivate medical talents with strong professional competency. How are we doing in this aspect?

**MF Li:** I think we should first clarify the connotation and extension of ‘professional competency’. Since the beginning of the twenty-first century, professional competency has been highlighted to be the major goal of medical education worldwide. As the COVID-19 pandemic exposed room for further improvement in medical education, we need to ask whether there is any overlooked aspect in our understanding of professional competency. For example, did this concept sufficiently emphasize the importance of humanistic and scientific spirit? Should we pay more attention to the competency in dealing with public-health knowledge and skills associated with infectious diseases? We should think about these questions and try to better recognize and define the criteria of professional competency.

**H Yan:** The professional competency of graduates reflects the quality of a medical school. If a medical school has high-quality teachers, facilities and affiliated hospitals, and if it is able to enroll high-quality students and cultivate them with well-designed teaching programs, then the professional competency of its graduates will surely meet the requirements of society.

In China, we have >200 medical schools at all levels, having their own features and aiming for different types of medical workers. We need world-class clinicians, medical scientists and medical educators. We also need grassroots medical workers to serve the primary hospitals in many counties and communities. Actually, the COVID-19 pandemic told us that grassroots medical workers are crucial for our healthcare system and we need a lot more of them.

The pandemic shows us the significance of medical education on national security. To strengthen medical education, we should continuously reform the very details of the system: better integration of basic, clinical and preventive medicine, improved course design that encourages case-oriented tutorial discussion, more attention on medical humanistic aspects, more interactions between teachers and students, and strengthening of instruction at all stages of medical education. These are the things we need to do in order to improve the professional competency of our students.

**HB Shen:** While rethinking issues concerning the pandemic, we often mention the cultivation of high-level interdisciplinary medical experts. First, we should help students to lay a solid foundation of basic knowledge and skills at their undergraduate stage. With this as the basis, we can further lead them to broaden

In China, we have >200 medical schools at all levels, having their own features and aiming for different types of medical workers.

—Hong Yan

their perspectives and improve their abilities of medical research and innovation.

Classified cultivation is a classic institutional design in medical education. Medical schools need to be classified into different types, so that some aim to cultivate high-level experts in specialties and some focus on training grassroots medical workers. On top of that, students in the same school should also be categorized according to their disciplines and interests, and trained in different categories with distinct curricula. For instance, for those students interested in scientific research with academic pursuit in mind, we can provide them with opportunities and platforms for innovative research training as early as the undergraduate stage. The same goes for graduate students. We should make individual approaches to train different categories of students, establishing different curricula and practice opportunities for each group.

**X Chen:** Maybe we should rephrase the expression of ‘cultivate high-level interdisciplinary talents’ because it is almost impossible to cultivate this kind of medical talent within 5 or 8 years of education. It is possible to cultivate ‘potential high-level interdisciplinary talents’. We hope the medical-school graduates can be ‘stem-cell-like talents’ that have the potential to differentiate into experienced clinical specialists, beloved primary-care physicians, trustable public-health experts or physician-scientists devoted to mechanistic studies of complex diseases.

The primary task of medical schools is to educate the students on how to be a qualified medical worker and how to be an honest and kindhearted human being. An excellent medical worker is not only someone who has superb clinical skills, but also someone who is brave, diligent, prudent and fair-hearted. That is what ancient Chinese philosopher Wang Yangming meant by ‘the unity of knowledge and action’. We should never forget this point when we are trying to foster potential high-level interdisciplinary medical talents.

**M Kuang:** Another question is: What kind of doctors should we cultivate for the future? According to the experiences against COVID-19, tomorrow's physicians should be able to not only cure diseases, but also deal with public-health emergencies. Some of them should also be excellent researchers for developing better treatments for multiple diseases. Moreover, besides clinical practitioners and researchers, they should also be good educators for the training of next-generation physicians.

To improve the quality of medical education fundamentally in China, faculty development is a priority. I have attended the annual conference of the Association for Medical Education in Europe since 2016 and noticed that ∼90% of the participants are clinicians. They consider medical education as the duty of all clinicians, not just medical-school administrators. Physicians themselves should be the major force to improve medical education in China. We should foster more talents who are good at both clinical practice and medical education. Maybe we can set up master's and doctor's programs as part of medical education and encourage clinicians to learn more about medical education during their continuing education.

**X Chen:** I think the ‘the last kilometer’ of the running course of teaching is to train qualified medical teachers. In the past 20 years, we have made a great effort to improve the quality of both medical research and clinical practice. But perhaps we should think more about how to give more respect and development space for medical teachers so that they would be glad to devote more effort into teaching and care more about the real development of the students.

## CONTINUING EDUCATION AFTER GRADUATION

**MF Li:** To maintain professional competency during one's professional career, all three stages of medical-school education, residency training and lifelong continuing medical education, as well as the continuum between these stages, should all be reinforced. In the medical-school stage, the students should learn enough about public health, preventive medicine and humanistic concerns. In the standardized training of resident physicians, trainees should be able to find their own balance of multidisciplinary clinical skills. In the continuing-education stage, maybe we should think about further strengthening the credit-earning mechanism, based on which a better requalification mechanism can be implemented for mid- and advanced-career physicians.

**XH Wan:** In this pandemic, relatively large-scale infection of medical workers occurred in Wuhan Central Hospital and several other hospitals. But the infection rate of respiratory- or infectious-disease-department physicians was relatively low. This implies that most physicians of other departments do not have the experience and skills to protect themselves from serious infectious diseases. Also, at the early stage of the pandemic, many physicians from other departments were not familiar with respiratory diseases and the success rate for emergency treatment of severe patients was relatively low. Considering these problems, I think we should propose more detailed official requirements for teaching the practical skills of treating infectious diseases in medical-school education.

Acute respiratory infectious diseases such as SARS, MERS, COVID-19 and influenza A are highly infectious, rapidly mutating and difficult to control. We should strengthen the education of these diseases so that our future physicians can better treat these patients, control the spread of the viruses and protect themselves.

One good news is that, on 2 March 2020, the Chinese government issued 16 new occupations and one of them is respiratory therapist. Respiratory therapists are specialists who offer multiple breathing-support and airway-management therapies to patients suffering respiratory insufficiency. That is what is needed for COVID-19 treatment. In this pandemic, >140 respiratory therapists went to Wuhan to help. Now, this occupation is officially recognized and there will be more qualified respiratory therapists and they will be a strong force to deal with future respiratory diseases.

Moreover, I have a suggestion for the continuing medical education. When I was studying in the USA in the 1990s, my mentor, a physician and psychiatrist, told me that, when the AIDS outbreak occurred in the USA, all American physicians of all departments had to learn about AIDS for at least two class hours to get the annual credit for their continuing education. I think we should learn from this strategy and require a minimum class hour of new infectious diseases and serious public-health events in our continuing medical education.

## MORE GENERAL PRACTITIONERS ARE NEEDED

**GQ Chen:** As you have mentioned, we need not only high-level interdisciplinary medical talents, but also general practitioners who can treat minor diseases, recognize serious diseases, transfer the serious patients to superior hospitals and manage chronic diseases competently. General practitioners are critical for our tiered system of medical service and for the rational use of our medical resources. However, we are currently weak in cultivating general practitioners.

**Y Ke:** More than 70% of diseases are age-related chronic diseases. These patients need long-term management by general practitioners in the primary hospitals, whose duty is to prevent, treat and follow up common diseases in the long term. On the other hand, the duty of tertiary hospitals is to treat difficult and severe diseases, to develop and implement new therapies, and to train young medical workers. This tiered system is essential for the treatment of different diseases, but does not mean that physicians in tertiary hospitals are more important than those in primary hospitals.

In the past, general practitioners mostly focused on common and chronic diseases, but this pandemic reminds us that they should also play an important role in treating infectious diseases. Most of the mild COVID-19 patients should have been diagnosed and treated in primary hospitals. However, without strong primary hospitals, all the patients were crowded into tertiary hospitals, occupying the medical resources for severe patients and even triggering further spread of the virus among the crowd. Many patients and their families had to wait anxiously for a long time in the hospital and the situation for some patients may have got worse during this waiting. We do have to promote and accelerate the establishment of a strong primary medical system, starting from the cultivation of general practitioners.

**BR Chi:** The pandemic exposed the problem that we have paid too much attention to specialized medicine education, but are weak in general-medicine education. China formally established the college major of general medicine in 2010 as a cross-disciplinary major combining clinical medicine, rehabilitation medicine, preventive medicine and health management. But the educational resource for general medicine remains insufficient and the educational system has not been standardized or

Now, we have only 2.2 general practitioners for every 10 000 residents.

—Baorong Chi

qualified. Now, we have only 2.2 general practitioners for every 10 000 residents. General practitioners are the first line to the prevention and control of both infectious and non-infectious diseases. We need to increase the population and better educate students who have majored in general practice with multiple practical skills.

**GQ Chen:** There is a famous Chinese quote that ‘erudition is the premise of becoming a doctor’. To cultivate medical talents—general practitioners or interdisciplinary talents—the most important thing is to cultivate students’ ability to active learning. They should not constrain themselves to the courses and tests. They should actively acquire knowledge and skills through all possible ways. That is the premise of cultivating great medical workers.

## MEDICAL EDUCATION AS AN IMPORTANT PART OF SOCIETY

**GQ Chen:** Health is the basis of a person and persons are the basis of a society. The pandemic told us again that the medical-care system is crucial for people's health, social stability, economic development and national security. It is important to build a social atmosphere that respects life and medicine.

**H Yan:** The pandemic forces us to reconsider the strategic position of medical education for our society. We hope that the whole of society would emphasize more about medical education and the government can invest more in medical education. Moreover, we need a better teacher-evaluation system, so that the medical teachers, especially the young teachers, would be more inclined to put more effort into the practice and innovation of medical education. That is one of the keys to ensuring the quality of medical teachers and, thus, the quality of medical education.

**Y Ke:** Medical education does not exist in isolation. If the medical-care system is not well built, it would be impossible for medical schools to attract best students and cultivate high-quality medical workers. So, on the one hand, we should call for society and the government to improve the entire medical system and, on the other, we educators should foresee the needs of our society and cultivate medical students who are prepared for the future. There are many things that can be done.

**X Chen:** In the coming post-pandemic era, we hope that the society can give medical workers and medical educators more understanding and kindness. Maybe we can establish a national multidisciplinary medical-education committee, which may help to improve the overall quality of medical education.

**MF Li:** In recent years, the public have conceptually agreed that the cost to educate and train a medical professional is high, but do not really recognize the serious consequences that would be caused by medical-education deficiencies. In fact, the investment for medical education is still far from enough. Many medical schools have to enroll more students in order to obtain sufficient finance from governments. The COVID-19 pandemic forces us to reconsider this situation. If we do not invest enough in medical education, the consequences may be impossible for our nation to bear.

The best way to foster students’ humanistic spirit is through the personal demonstration of the teachers.

—Yang Ke

## CULTIVATE STUDENTS’ RESPONSIBILITY AND HUMANISTIC SPIRIT

**GQ Chen:** We need social recognition and national investment. But, actually, it is difficult to change society. All we can do is to change ourselves. It is always important to improve the social responsibility of medical teachers and students.

**YQ Hu:** The 2020 COVID-19 pandemic swept across the globe, impacting almost every country in the world. Tens of thousands of Chinese medical workers resolutely headed to the front line, successfully saved many lives and controlled the pandemic within a short time. The whole of society is greatly touched by the strong social responsibility of the medical workers and began to think about what are good medical workers and how to cultivate qualified medical workers with strong humanistic spirit in the future. We talked much about these questions in our discussion. We should further combine the forces of hospitals and medical schools, and reinforce the connection between medical practice and medical education. We should also emphasize the multidisciplinary nature of medicine throughout the entire medical-education process from medical schools to graduate medical education and continuing education.

**Y Ke:** There are two major requirements for clinical talents. The first is their ability to cure patients, or their clinical competency. This ability is reflected not by how they are doing in the tests or how much knowledge they have memorized in their minds, but by their ability to diagnose and treat complex clinical cases—not only the diseases of a particular discipline, but also infectious diseases or other diseases when needed. This ability cannot be obtained only through classroom teaching. So one of the directions of medical-education reform is to generally teach the basic concepts and knowledge, and then start practical teaching as early as possible. The students can learn about the detailed medical knowledge, and also develop their abilities to solve practical problems and to communicate with colleagues and patients through the practical-teaching process.

The second requirement is the humanistic spirit of medicine. Clinical work requires strong kindness, compassion and empathy. Excellent physicians can explain the condition of illness to the patients, comfort their anxiety and make up the best treatment plan from the perspective of patients in a good way. The best way to foster students’ humanistic spirit is through the personal demonstration of the teachers. For example, in this pandemic, the socially responsible behaviors of the elder medical workers greatly influenced many medical students. Besides, it is also important to integrate humanistic spirit into the evaluation criteria of medical workers. Good behaviors should be regularly rewarded.

**MF Li:** The humanistic spirit of medicine includes at least three aspects—the pursuit and upholding of the truth, the willingness and wish to contribute caregiving to human life in need, and the ability to light up human lives with the beauty of human civilization. We should pass the truth, kindness and beauty of medicine on to the next generation through all possible routes, including but not limited to courses, personal demonstrations and the professional environment.

**X Chen:** We all understand the importance of medical humanistic education, but the problem is how to realize it well. In a recent online conference held by the Chinese Ministry of Education, first-line medical educators talked about their experience of how to improve the quality of ideological and political courses. That has some reference values for our future work.

**BR Chi:** I would like to end this panel discussion with the ending of an article published in *Guangming Daily* by Prof. Guoqiang Chen:

The road of medicine is long and obstructed, which needs unremitting efforts and long persistence. The best and only choice for us medical educators should be: never give up, take responsibility and continuously adjust our direction towards the right way.

## SUMMARY

**GQ Chen:** Many thanks to Prof. Chi and all experts in the discussion. The pandemic has manifested the importance of the medical-care system. As medical workers, to appropriately react to the big changes that could appear, represented by the COVID-19 pandemic, we have to clearly recognize and scientifically cope with the situation and then actively peruse the solutions. Medical education shares fate with our nation and people. It will surely achieve fast development and contribute more to the health of Chinese people and the rejuvenation of China.

